# The genetic code of bipolar disorder: new frontiers in pharmacogenetics

**DOI:** 10.1192/j.eurpsy.2026.10343

**Published:** 2026-07-31

**Authors:** S. Papiol

**Affiliations:** Clinical Translation, Max Planck Institute of Psychiatry, Munich, Germany

## Abstract

**Abstract:**

Lithium remains the most effective mood stabilizer for bipolar disorder (BD), significantly reducing suicide risk and overall mortality. However, treatment response is highly heterogeneous and heritable (SNP-h² = 0.22–0.29), with familial clustering observed. The Consortium on Lithium Genetics (ConLiGen) was established to identify genetic determinants of lithium response able to disentangle such heterogeneity in lithium response.

ConLiGen Phase 1 conducted a genome-wide association study (GWAS) of 2,563 BD patients from 22 sites, evaluating lithium response both dichotomously and continuously via the Alda Scale. Four linked polymorphisms on chromosome 21 met genome-wide significance criteria for association with lithium response. The response-associated region contains two genes for long, non-coding RNA. ConLiGen Phase 2 is expanding recruitment to new sites, targeting > 6,000 participants, mainly including European cohorts.

We will present the results of Phase 1 ConLiGen study and also interim results adding new Phase 2 cohorts to the study. Likewise, we will also present ongoing analyses that use genetic information to define clusters of patients mapping to response/non-response phenotype.

ConLiGen Phase 2 will substantially increase statistical power and global representation to unravel the genetic basis of lithium response. This collaborative framework will accelerate discovery for personalized BD treatment and suicide prevention. Data sharing and new partnerships are actively encouraged.

**Disclosure of Interest:**

None Declared

